# Cell-biological effects of zinc oxide spheres and rods from the nano- to the microscale at sub-toxic levels

**DOI:** 10.1007/s10565-020-09571-z

**Published:** 2020-11-17

**Authors:** M. Olejnik, M. Kersting, N. Rosenkranz, K. Loza, M. Breisch, A. Rostek, O. Prymak, L. Schürmeyer, G. Westphal, M. Köller, J. Bünger, M. Epple, C. Sengstock

**Affiliations:** 1 Inorganic Chemistry and Center for Nanointegration Duisburg-Essen (CeNIDE), University of Duisburg-Essen, Essen, Germany; 2 Bergmannsheil University Hospital/Surgical Research, Ruhr-University Bochum, Bochum, Germany; 3 Institute for Prevention and Occupational Medicine of the German Social Accident Insurance, Institute of the Ruhr-University Bochum (IPA), Bochum, Germany

**Keywords:** Zinc oxide, Nanoparticles, Microparticles, Particle size, Particle shape, Inflammation, NR8383 alveolar macrophages, Particle-induced cell migration assay

## Abstract

Zinc oxide particles were synthesized in various sizes and shapes, i.e., spheres of 40-nm, 200-nm, and 500-nm diameter and rods of 40∙100 nm^2^ and 100∙400 nm^2^ (all PVP-stabilized and well dispersed in water and cell culture medium). Crystallographically, the particles consisted of the hexagonal wurtzite phase with a primary crystallite size of 20 to 100 nm. The particles showed a slow dissolution in water and cell culture medium (both neutral; about 10% after 5 days) but dissolved within about 1 h in two different simulated lysosomal media (pH 4.5 to 4.8). Cells relevant for respiratory exposure (NR8383 rat alveolar macrophages) were exposed to these particles in vitro. Viability, apoptosis, and cell activation (generation of reactive oxygen species, ROS, release of cytokines) were investigated in an in vitro lung cell model with respect to the migration of inflammatory cells. All particle types were rapidly taken up by the cells, leading to an increased intracellular zinc ion concentration. The nanoparticles were more cytotoxic than the microparticles and comparable with dissolved zinc acetate. All particles induced cell apoptosis, unlike dissolved zinc acetate, indicating a particle-related mechanism. Microparticles induced a stronger formation of reactive oxygen species than smaller particles probably due to higher sedimentation (cell-to-particle contact) of microparticles in contrast to nanoparticles. The effect of particle types on the cytokine release was weak and mainly resulted in a decrease as shown by a protein microarray. In the particle-induced cell migration assay (PICMA), all particles had a lower effect than dissolved zinc acetate. In conclusion, the biological effects of zinc oxide particles in the sub-toxic range are caused by zinc ions after intracellular dissolution, by cell-to-particle contacts, and by the uptake of zinc oxide particles into cells.

**Figure:**
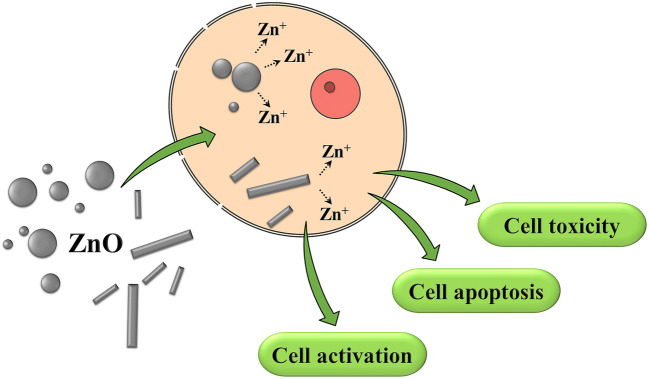
**Graphical headlights** • The cytotoxicity of zinc oxide particles is mainly due to the intracellular release of zinc ions. • The size and shape of zinc oxide micro- and nanoparticles has only small effects on lung cells in the sub-toxic range. • Zinc oxide particles are rapidly taken up by cells, regardless of their size and shape. • Zinc oxide particles rapidly dissolve after cellular uptake in endolysosomes.

**Graphical headlights**

• The cytotoxicity of zinc oxide particles is mainly due to the intracellular release of zinc ions.

• The size and shape of zinc oxide micro- and nanoparticles has only small effects on lung cells in the sub-toxic range.

• Zinc oxide particles are rapidly taken up by cells, regardless of their size and shape.

• Zinc oxide particles rapidly dissolve after cellular uptake in endolysosomes.

## Introduction

Health risks from particles and fibers are still a highly topical challenge for health protection at the workplace. Scientific studies suggest that the particle surface has a major influence on their harmful effects on health. However, there are still unanswered questions regarding the prevention of particle-related respiratory diseases. Crucial open questions concern the influence of particle size, particle shape, and particle surface area on biological effects and potential health risks. Particle-induced diseases represent a large proportion of occupational diseases, and despite many years of research in this field, there are still unresolved issues for the prevention of particle-induced respiratory disorders. In addition to acute effects such as coughing, lacrimation, and irritation of the upper respiratory tract, a long-term exposure to particulate matter is of high relevance. Health hazards caused by inhalation of particles including fibers are usually due to their inflammatory properties (Pietroiusti et al. 2018; Borm et al. 2018; Kawasaki 2015; Landsiedel et al. 2012, 2014a, b; Donaldson et al. 2013; ; Klein et al. 2012; Paur et al. 2011; Seaton et al. 2010). For instance, welders and galvanizing workers can be affected by a transient metal fume fever, the so-called zinc fume fever. Indeed, animal inhalation experiments have shown a strong toxicity of welding fumes when zinc was present and not just iron (Antonini et al. 2017). The systemic induction of metal fume fever discriminates ZnO from insoluble particles that predominantly induce local inflammatory effects in the lung. In fact, ZnO is slightly soluble in water, and animal inhalation studies suggest that these systemic effects are related to the release of Zn^2+^ ions. This was concluded because mass concentration and surface area were correlated with the ZnO toxicity rather than the particle concentration (Ho et al. 2011).

Landsiedel et al. performed in vivo inhalation studies with rats using nano-ZnO and micro-ZnO (both highly agglomerated; 0.5 to 50 mg m^−3^) for 5 days and analyzed the response after 14 and 21 days. They observed a temporary body weight gain and pulmonary inflammation for both sizes. For micron-sized ZnO only, they observed an increase in lung weight. They concluded that 14 or 21 days may not be sufficient to recover from the exposure to ZnO particles, probably due to the irreversible dissolution of zinc oxide and the resulting presence of zinc ions (Landsiedel et al. 2014a). Acute effects of ZnO were also investigated in a controlled human exposure study (inhalation): ZnO in concentrations of 0.5, 1.0, and 2.0 mg m^−3^ caused a dose-dependent induction of ZnO fever in a part of the volunteers, accompanied by an increase of neutrophilic granulocytes (Monsé et al. 2018, 2020a, b). No effects were observed for the cardiovascular system (Aweimer et al. 2020). Local irritative effects of ZnO on the respiratory system were reported after analyzing the induced sputum. Airway inflammation led to an increase of neutrophils, IL-8, IL-6, MMP-9, and TIMP-1 at a ZnO concentration of 0.5 mg m^−3^ (Monsé et al. 2019).

In general, insoluble particles act as local irritants and cause acute symptoms such as coughing, lacrimation, and irritation of the upper respiratory tract after acute exposure (Landsiedel et al. 2014a). Continuous high levels of exposure lead to chronic inflammation and as a consequence to diseases that are driven by chronic inflammation, such as chronic obstructive pulmonary disease (COPD), or even cancer. Since ZnO is only slightly soluble at neutral pH, such local inflammatory effects can occur as well. Consequently, the biological effects of ZnO will depend on the particle geometry, i.e., size and shape, which also affect the release rate of zinc ions (Singh et al. 2019; Mohajerani et al. 2019; Jeevanandam et al. 2018; Epple 2018; Chen et al. 2018; David et al. 2016; Shin et al. 2015). Whereas the particle size has been varied in many studies, the effect of particle shape has less frequently been studied in a systematic way.

Apart from their unintended presence in the workplace and the environment, zinc oxide particles have many applications in biomedical materials technology, nanomedicine, health care, and consumer products, where the particle parameters play the key role with respect to their physicochemical and biological properties (Ann et al. 2015; Cheng et al. 2013). There are different synthetic methods for the preparation of ZnO particles with defined properties, including chemical vapor deposition, sol-gel syntheses, hydrothermal synthesis, and precipitation methods (Amarilio-Burshtein et al. 2010; Dejene et al. 2011). Different strategies for the synthesis of ZnO particles in the microscale were reported, but only a few permit the synthesis of monodisperse ZnO particles of different shape in the size range between 50 and 500 nm in a well-dispersed, colloidally stable form (Eskandari et al. 2009; Nagvenkar et al. 2016).

Our aim was to prepare monodisperse and well-characterized ZnO particles in two different shapes (spheres and rods) in the size range from nano to micro with an in-depth chemical and crystallographic characterization. They were all functionalized with the same polymer, i.e., poly(N-vinyl pyrrolidone) (PVP), to have the same surface chemistry. Their sub-toxic biological effects were analyzed by high-end in vitro cell culture studies. Rather than determining cytotoxic concentrations (which were already reported earlier), we wanted to elucidate whether macrophages were activated, leading to a sub-toxic but possibly detrimental long-term inflammation. As outlined above, the local irritant ZnO can induce systemic pyrogenic effects after inhalation (Monsé et al. 2018; Landsiedel et al. 2014a; Monsé et al. 2019; Wang et al. 2019); therefore, a prediction of the sub-toxic effects can help to understand this effect from the viewpoint of occupational health safety.

## Results

Zinc oxide particles were prepared in two different morphologies (spheres and rods) and in different size ranges (from the nanoscale to the microscale). ZnO microparticles were prepared by hydrolysis of zinc nitrate in dimethylformamide (DMF) in a one-pot synthesis. ZnO nanoparticles were prepared by hydrolysis in high-boiling polyol solvents (ethylene glycol, diethylene glycol) in the presence of PVP (Lee et al. 2008). The stabilizer PVP served both to control the desired particle shape and to ensure the dispersibility of the particles after purification. Thus, all particles were PVP-coated. The particles were comprehensively characterized by scanning electron microscopy (SEM), dynamic light scattering (DLS), thermogravimetric analysis (TGA), UV/vis spectroscopy, infrared (IR) spectroscopy, and X-ray powder diffraction (XRD).

**Fig. 1:**
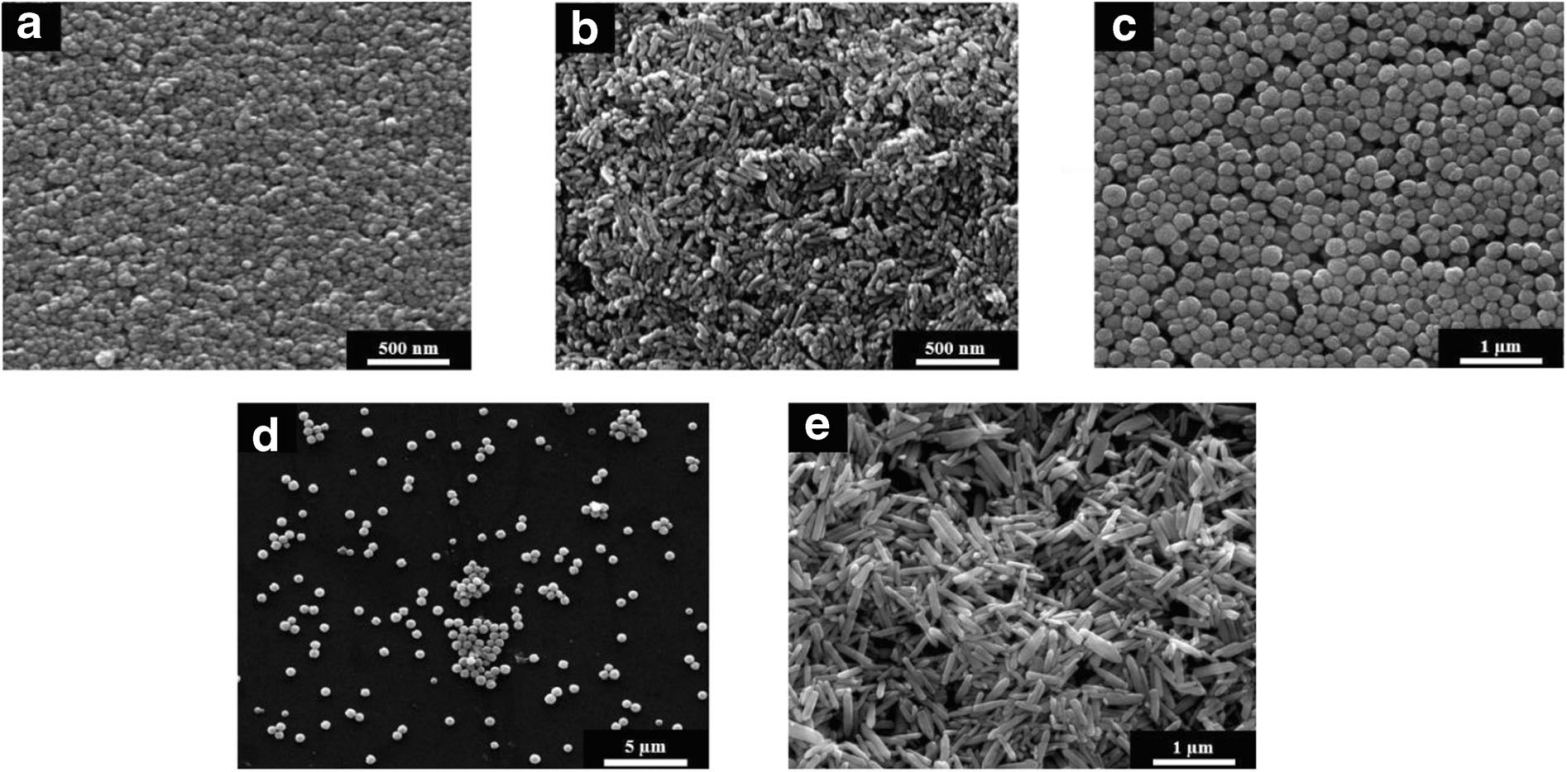
Scanning electron micrographs of ZnO particles of different size and shape: nanospheres (**a**), nanorods (**b**), submicrospheres (**c**), microspheres (**d**), and microrods (**e**)

**Fig. 2:**
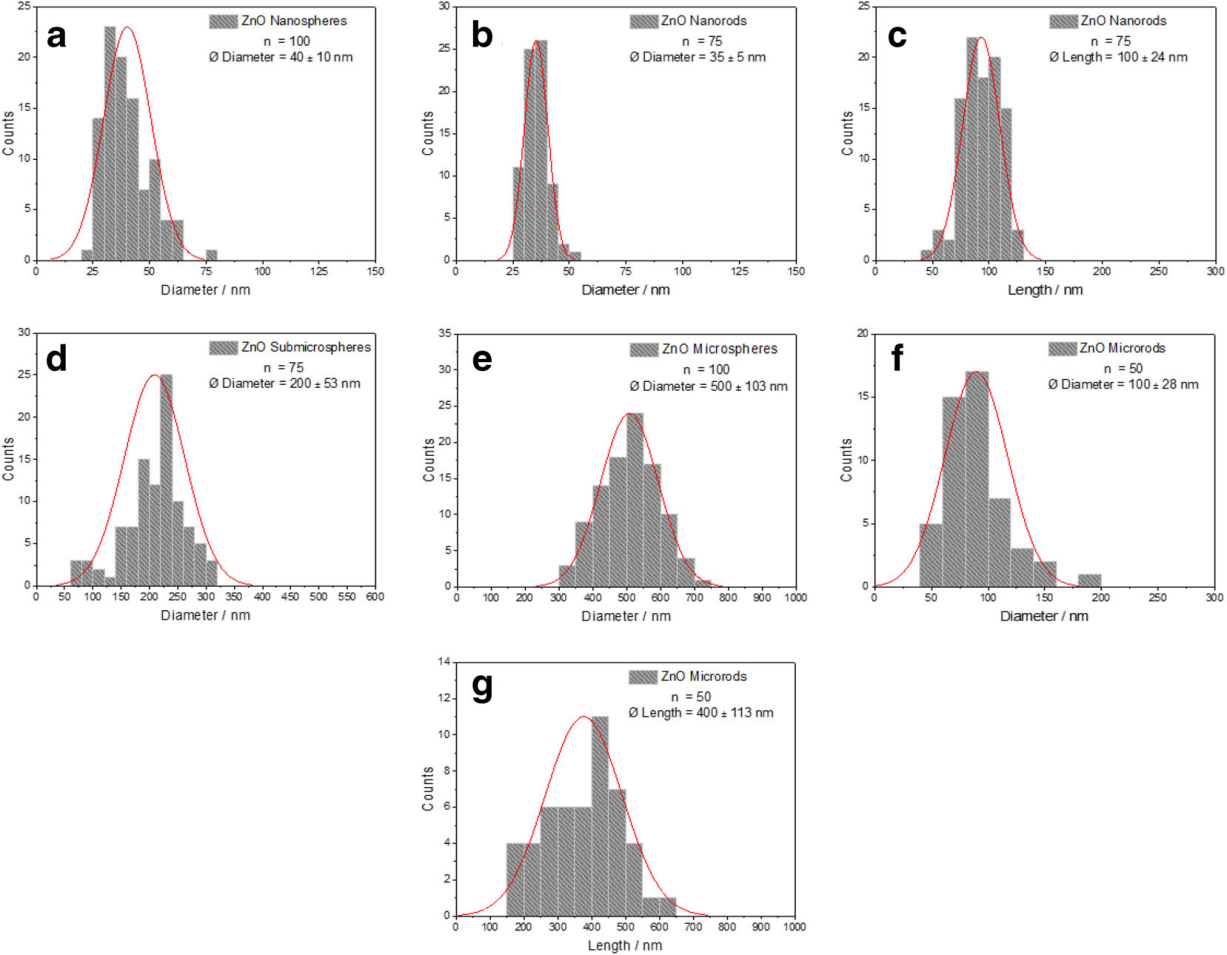
Particle size distribution (log-normal) of ZnO particles by SEM: diameter of nanospheres (**a**), diameter of nanorods (**b**), length of nanorods (**c**), diameter of submicrospheres (**d**), diameter of microspheres (**e**), diameter of microrods (**f**), length of microrods (**g**)

The size of the ZnO particles and their morphology were determined by SEM (Fig. 1). Scanning electron micrographs showed monodisperse, rod-like particles and almost spherical particles with a narrow particle size distribution. The corresponding histograms of the number-weighted particle diameters and lengths are given in Fig. 2.

**Fig. 3:**
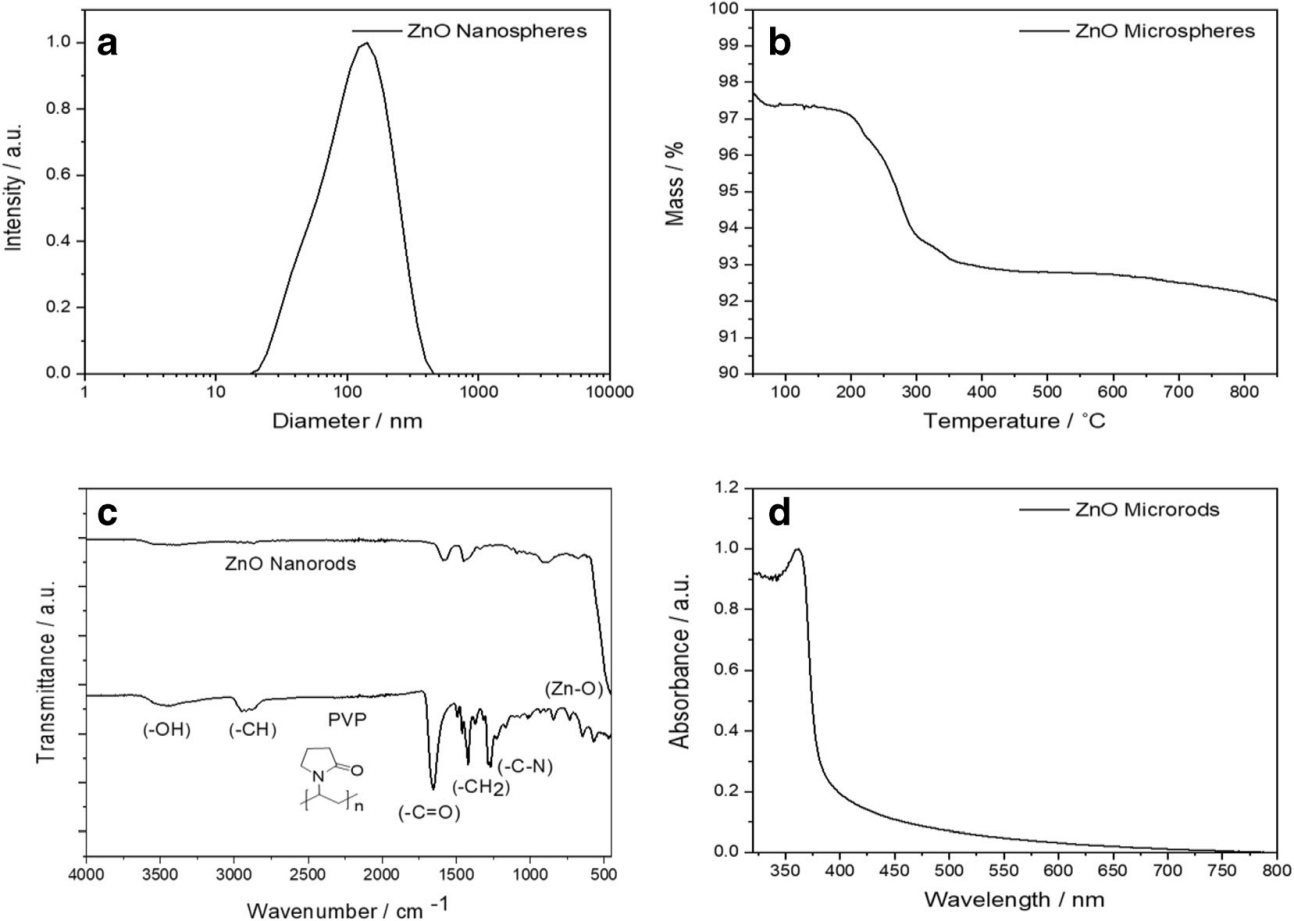
Representative characterization data of PVP-functionalized ZnO particles: Dynamic light scattering (DLS) (**a**), thermogravimetry (TG) (**b**), IR spectroscopy of PVP-functionalized zinc oxide nanorods and of pure PVP (**c**), and UV/vis spectroscopy (**d**). The other particle morphologies gave similar results

Figure 3 shows representative characterization data of the synthesized ZnO particles. To investigate the colloidal stability and the size distribution of the dispersed particles, DLS was performed. The polydispersity index (PDI) was below 0.4 for all types of ZnO particles, indicating a low agglomeration tendency of the particles in water, supported by the good agreement between hydrodynamic diameter by DLS and core diameter by SEM. To determine the amount of the polymer PVP on the particle surface, the particles were analyzed by thermogravimetric analysis in oxygen atmosphere. The combustion of PVP occurred between 200 and 400 °C. In addition to the TG measurements, the particles were analyzed by IR spectroscopy for the presence of PVP on the particle surface. The characteristic strong vibrational modes of the ZnO lattice at 452 cm^−1^, of the methylene group at 1408 cm^−1^, and the stretching vibration of the carbonyl group at 1651 cm^−1^ were all found in the IR spectrum (Bellamy 1975). The UV/Vis spectrum showed a broad absorption peak with an intensity maximum at 360 nm for all samples. The profile of the absorption peak is typical for ZnO nanoparticles in aqueous dispersion and due to its semiconductor bandgap (Marczak et al. 2009). All physicochemical particle parameters are summarized in Table 1.

**Table 1:** Physicochemical characterization data of all synthesized ZnO particles

System	Particle size by SEM/nm	Hydrodynamic diameter by DLS/nm	PDI from DLS	Zeta-potential by DLS/mV	polymer content by TG/wt%	Particle number per g solid (calculated)	Specific surface area (calculated)/m^2^ g^−1^
Nanospheres	40	101	0.351	+ 23	1.3	7.40 × 10^15^	37
Nanorods	40∙100	130	0.217	− 15	0.8	1.98 × 10^15^	27
Submicrospheres	200	254	0.101	+ 12	2.4	5.12 × 10^13^	8.2
Microspheres	500	525	0.086	− 15	3.9	3.79 × 10^12^	3.0
Microrods	100∙400	601	0.216	− 10	3.3	7.82 × 10^13^	10

**Fig. 4:**
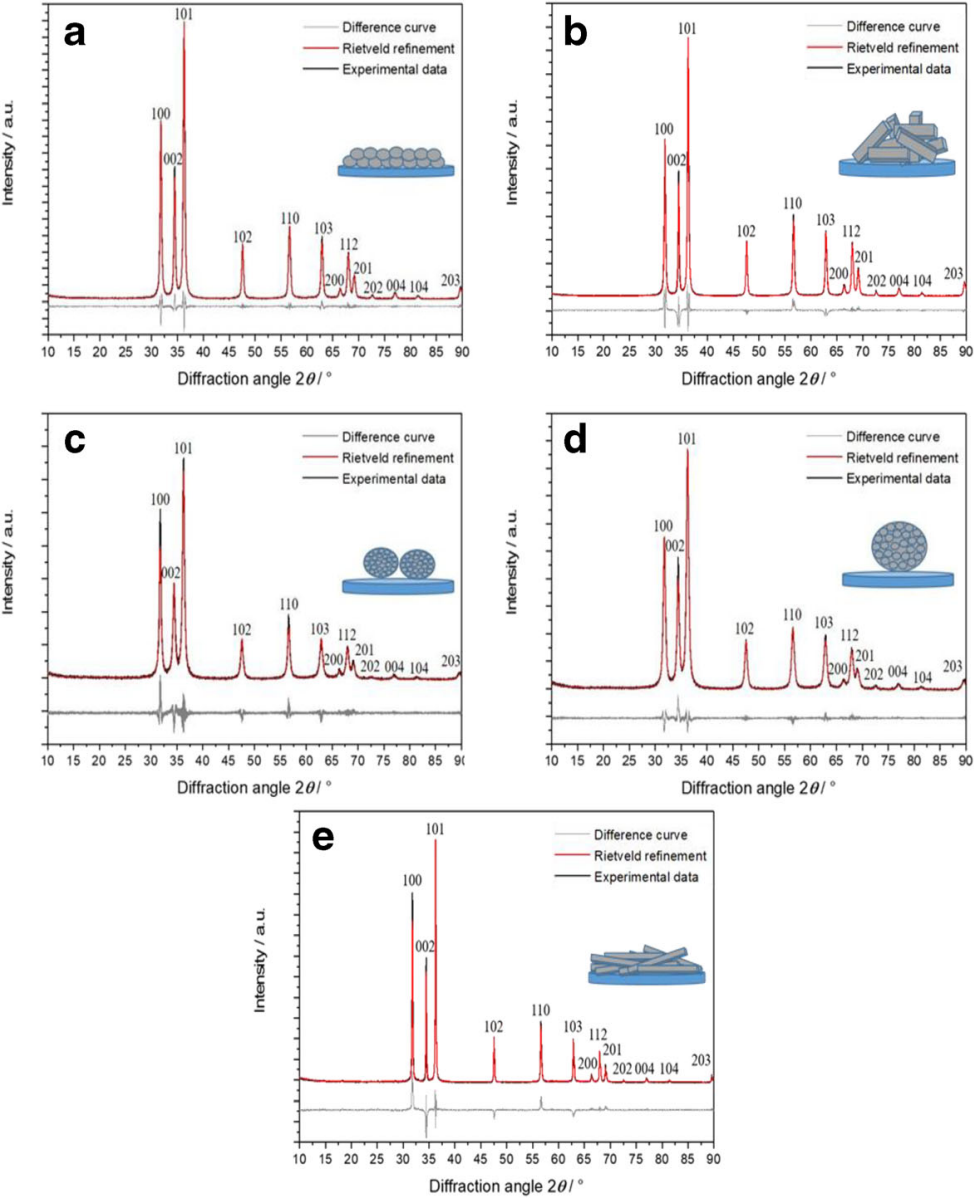
X-ray powder diffractograms of ZnO nanospheres (**a**), ZnO nanorods (**b**), ZnO submicrospheres (**c**), ZnO microspheres (**d**), and ZnO microrods (**e**)

The samples were investigated by XRD and analyzed by quantitative Rietveld refinement to determine their crystallographic properties and phase purity (Fig. 4). Note that ZnO can crystallize both in cubic (sphalerite or zinc blende) and in hexagonal (wurtzite) forms which have different physicochemical (Zagorac et al. 2014) and possibly also biological properties. In all cases, the pattern showed the diffraction peaks of the wurtzite lattice of hexagonal ZnO (space group *P*6_3_mc). No crystalline impurities were detected. The peaks of the elongated nano- and microrods had a different width, i.e., sharper for [00l] and broader for [h00], depending on the crystal size (Table 2).

**Table 2:** X-ray powder diffraction (XRD) data for PVP-stabilized ZnO particles for different lattice planes (hkl) by Rietveld refinement. *a* and *c* lattice parameters of the hexagonal unit cell; *ε* microstrain; *D* crystallite size

	Nanospheres	Nanorods	Submicrospheres	Microspheres	Microrods
*a*/Å	3.2518 (1)	3.2513 (1)	3.256 (1)	3.253 (1)	3.2519 (1)
*c*/Å	5.2085 (2)	5.2084 (2)	5.215 (2)	5.210 (1)	5.2079 (1)
*ε*/%	0.06 (1)	0.04 (1)	0.09 (1)	0.01 (1)	0.02 (1)
Average *D*/nm	33 (1)	44 (1)	21 (1)	20 (1)	107 (2)
(hkl)	Anisotropic crystallite size (*D*_A_)				
(100)	29	35	25	20	93
(002)	37	70	16	24	274
(101)	30	39	21	21	104
(102)	32	45	17	21	114
(110)	31	39	23	20	92
(103)	35	51	16	21	133
(200)	29	35	20	18	72
(112)	31	42	18	19	96
(201)	31	40	21	18	82
(004)	37	71	15	19	213
(202)	32	40	16	20	98
(104)	36	53	14	18	147
(203)	26	40	20	24	100

This dependency was clearly shown by the determination of anisotropic crystallite size (*D*_A_) in agreement with the electron microscopic data (Fig. 1). In contrast to this, the spherical particles (nano, submicro, and micro) showed an isotropic crystallite size (30, 21, and 20 nm, respectively) which was reflected by comparably broad peak profiles for all crystallographic planes (hkl). Based on the XRD and SEM results, we conclude that the nanospheres and nanorods were single-crystalline, whereas the submicrospheres and the microspheres were polycrystalline.

**Fig. 5:**
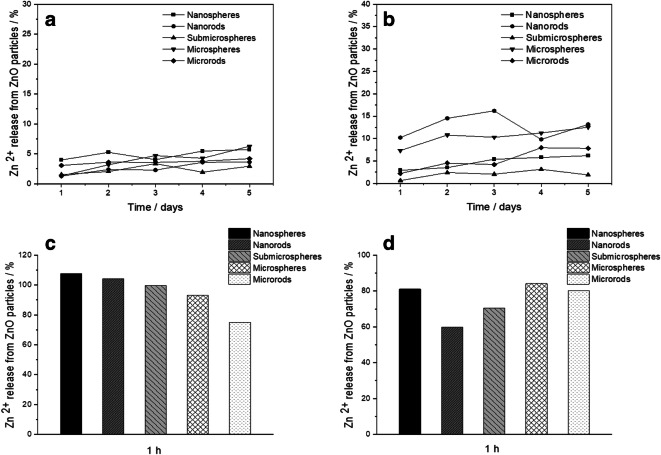
Dissolution data of ZnO particles at 37 °C: in water (**a**), in RPMI medium supplemented with 10% FBS (**b**), in simulated lysosomal medium for 1 h (**c**), and in simulated citrate-free lysosomal medium (acetate buffer) for 1 h (**d**)

ZnO is considered soluble material according to EU regulations (EFSA 2016). Zinc ions from the dissolution of zinc oxide have been proposed as the major source of the biological action of ZnO nanoparticles (Liu et al. 2016; Ziglari et al. 2020). Chemically, zinc oxide is sparingly soluble but not completely insoluble in water. Therefore, we have measured the release of zinc ions (Zn^2+^) from the prepared ZnO particles both in water and in cell culture medium (RPMI+10% FCS). Figure 5 shows the dissolution kinetics. The dissolution in cell culture medium was slightly faster (5 to 15%) than the dissolution in pure water (< 10%), possibly due to the complexation of zinc ions by proteins and other biomolecules in the cell culture medium. However, no significant differences in the ion release rate were found for different particle sizes and shapes, despite the different specific particle surface area (Table 1).

It is generally accepted that particles end up in acidic cell organelles like endosomes, endolysosomes, and lysosomes (endo-/lysosomes) after cellular uptake (Nazarenus et al. 2014; Kuhn et al. 2014; Canton and Battaglia 2012; Sahay et al. 2010). There, a low pH around 4 is prevalent which can lead to the dissolution of nanoparticles (Patel et al. 2019). Therefore, we have also studied the dissolution rate in a simulated lysosomal medium (Henderson et al. 2014). It turned out that the dissolution was much faster (duration 1 h or less), first due to the low pH, but probably also enhanced by the presence of large amounts of citrate (buffering compound) that can dissolve ZnO by complexation of Zn^2+^. Thus, we repeated the dissolution experiment in citrate-free acetate-buffered medium. In this medium, the dissolution was somewhat slower but still remarkably faster than in water or cell culture medium, pointing to the effect of a low pH. There was no significant difference between the different particle types, i.e., the specific surface area did not play a major role in the dissolution kinetics.

**Fig. 6:**
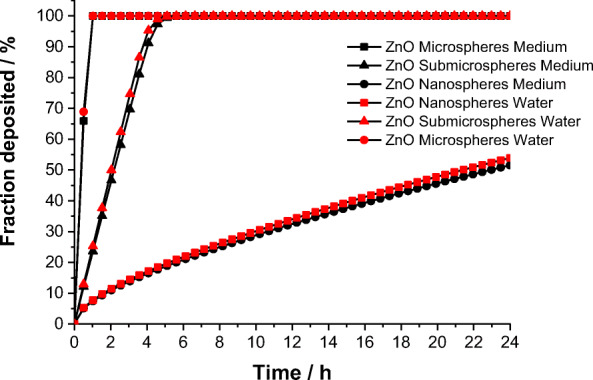
Precipitation and diffusion of ZnO particles according to the ISDD model (Thomas et al. 2018) in water and in cell culture medium

As typical cells of the immune system, NR8383 rat alveolar macrophages were used for cell culture experiments and incubated with ZnO particles in defined doses with different specific particle surface areas (Table 3). The cell-to-particle contact plays a strong role in the biological effects (Feliu et al. 2017; Nazarenus et al. 2014). Figure 6 shows the computed sedimentation rate of the ZnO particles according to the in vitro sedimentation, diffusion, and dosimetry model (ISDD) (Thomas et al. 2018). Due to their high density, the microparticles will sediment rapidly onto the cells, whereas the nanoparticles remain in dispersion for a much longer time. However, this model does not consider any dissolution of ZnO particles, neither before nor after cellular uptake.

**Table 3:** Dose data for cell culture studies with different ZnO particles, computed for 100 mg particles L^−1^ in a 24-well plate (2 cm^2^, 640 uL medium, 48,000 cells)

System	Zinc oxide concentration /mmol L^−1^	ZnO particle concentration/L^−1^	ZnO particle surface/m^2^ particle	ZnO particle surface/m^2^ L^−1^	ZnO concentration/particles m^2^
Nanospheres	1.23	7.41 × 10^14^	5.03 × 10^−15^	3.72	2.37 × 10^15^
Nanorods	1.23	1.97 × 10^14^	1.38 × 10^−14^	2.73	6.32 × 10^14^
Submicrospheres	1.23	5.92 × 10^12^	1.38 × 10^−13^	0.82	1.90 × 10^13^
Microspheres	1.23	3.79 × 10^11^	7.85 × 10^−13^	0.29	1.21 × 10^12^
Microrods	1.23	6.32 × 10^12^	1.65 × 10^−13^	1.04	2.02 × 10^13^

**Fig. 7:**
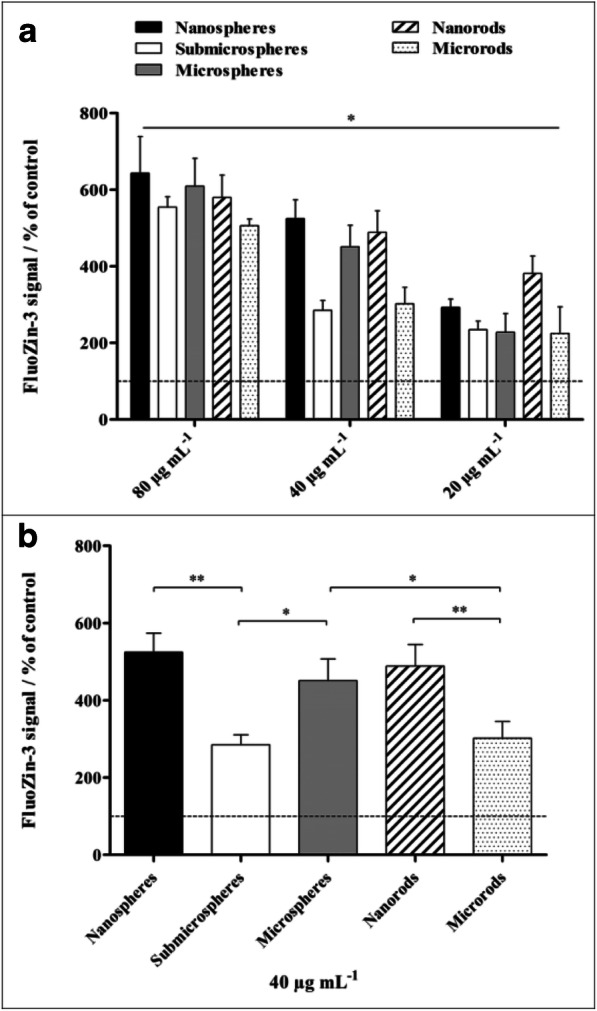
Intracellular zinc ion concentration in NR8383 alveolar macrophages after 2 h of exposure to different ZnO particles determined by flow cytometry. Zinc ions were quantitatively detected by the Zn^2+^-selective indicator FluoZin-3. **a** Comparison of the effects of different particle concentrations and morphologies. **b** Comparison of the effects of different particle morphologies at a concentration of 40 μg mL^−1^. All concentrations refer to solid ZnO. The data are expressed as mean ± SD (*N* = 3), given as the percentage of the control (100%, untreated cells). Asterisks (*) indicate significant differences in comparison to the control (*p ≤ 0.05, **p ≤ 0.01)

The uptake of ZnO particles by NR8383 cells was not detectable by focused ion beam milling and transmission electron microscopy with energy-dispersive X-ray spectroscopy as shown earlier for silver nanoparticles (Greulich et al. 2011), possibly due to the rapid intracellular dissolution. ZnO nanoparticles are only weakly autofluorescent. As we did not want to add a fluorescent label in order to avoid a change in the biological response, the particle uptake was studied indirectly by measuring the Zn^2+^ release after cellular uptake. The intracellular Zn^2+^ concentration in NR8383 cells after 2 h of exposure to different ZnO particles was determined by flow cytometry with the Zn^2+^-selective indicator FluoZin-3 (Fig. 7). We found a concentration-dependent effect for all particle types, as the intracellular Zn^2+^ concentration increased with the concentration of the particles, as expected. Particle size-dependent effects were observed at 40 μg mL^−1^ for submicrospheres, for which a significantly lower Zn^2+^ concentration was detected compared with nano- and microspheres. A significant reduction was also observed for microrods compared with nanorods. Differences due to morphology were detected only between microspheres and microrods at 40 μg mL^−1^.

**Fig. 8:**
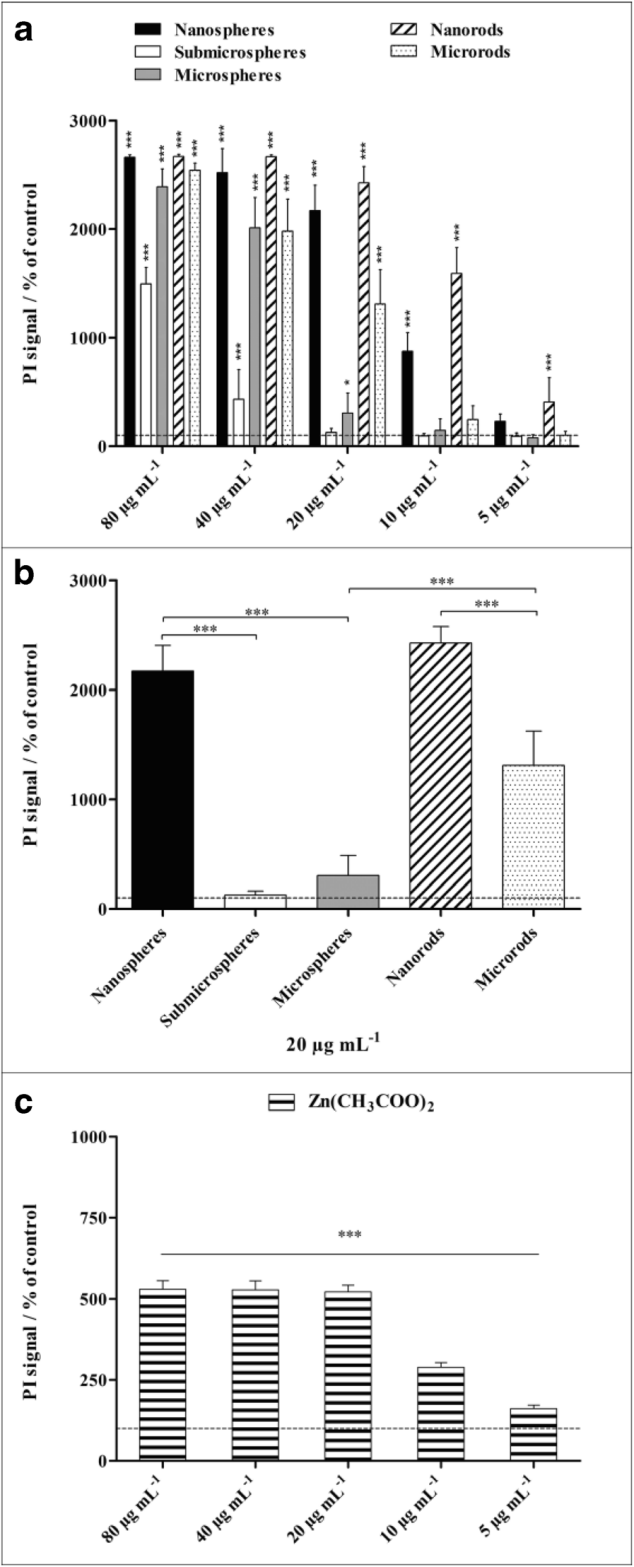
Viability of NR8383 alveolar macrophages after 16 h of exposure to different ZnO particles (**a**, **b**) and to a zinc acetate solution as control (**c**). The cell viability was determined by propidium iodide (PI) staining of non-viable cells, and the mean PI fluorescence intensity was assessed by flow cytometry. **a** Comparison of the effects of different particle concentrations and morphologies. **b** Comparison of the effects of different particle morphologies at a particle concentration of 20 μg mL^−1^. All concentrations refer to solid ZnO, except for zinc acetate where the concentration of Zn^2+^ is given. The data are expressed as mean ± SD (*N* = 3), given as the percentage of the control (100%, untreated cells). Asterisks (*) indicate significant differences in comparison to the control (*p ≤ 0.05, ***p ≤ 0.001)

Propidium iodide (PI) staining of non-viable cells was used to determine the cytotoxicity of different ZnO particles on NR8383 cells after 16 h of exposure (Fig. 8). All ZnO particle types had significant cytotoxic effects compared with untreated control cells at particle concentrations of 80 and 40 μg mL^−1^. Submicrospheres showed no cytotoxicity below 40 μg mL^−1^, and microspheres as well as microrods were non-toxic below 20 μg mL^−1^. Thus, nanoparticles resulted in the highest cell toxicity. In general, the cell viability was affected more strongly by rod-shaped particles compared with spherical particles of similar size. For instance, nanorods were more toxic than nanospheres at 10 and 5 μg mL^−1^, and microrods were more toxic than microspheres at 20 μg mL^−1^.

**Fig. 9:**
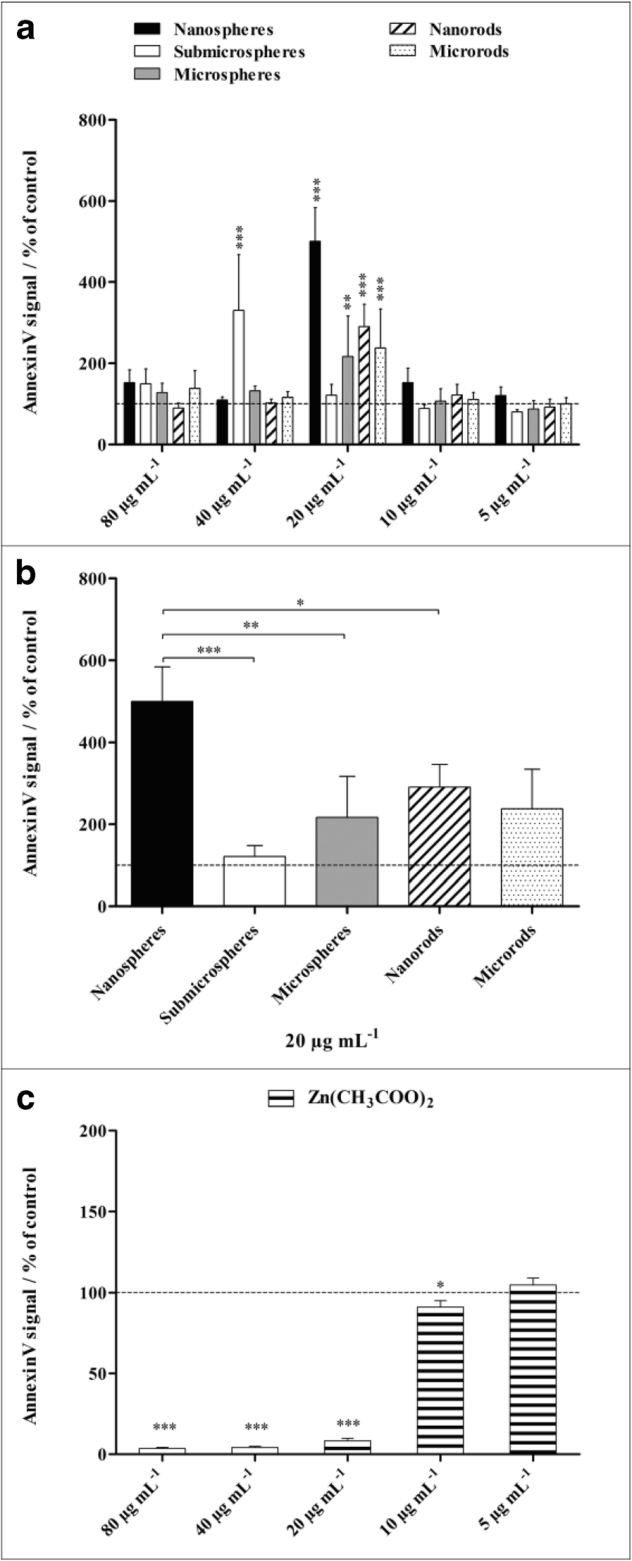
Induced apoptosis of NR8383 alveolar macrophages after 16 h of exposure to different ZnO particles (**a**, **b**) and a zinc acetate solution as control (**c**), analyzed by flow cytometry. Cells of early apoptosis were detected by FITC-conjugated Annexin V, and necrotic cells and cells of late apoptosis were excluded by PI staining. **a** Comparison of the effects of different particle concentrations and morphologies. **b** Comparison of the effects of different particle morphologies at a particle concentration of 20 μg mL^−1^. All concentrations refer to solid ZnO, except for zinc acetate where the concentration of Zn^2+^ is given. Data are expressed as mean ± SD (*N* = 3), given as the percentage of the control (100%, untreated cells). Asterisks (*) indicate significant differences in comparison to the control (*p ≤ 0.05, **p ≤ 0.01, ***p ≤ 0.001)

In addition to the cytotoxicity, the induction of apoptosis in NR8383 cells was investigated with the Annexin V assay. To exclude necrotic cells and cells of late apoptosis, a PI counterstaining was performed as well (Fig. 9). All ZnO particle morphologies led to a significant enhancement of apoptosis at a particle concentration of 20 μg mL^−1^, except for submicrospheres which induced apoptosis at 40 μg mL^−1^. Among the examined ZnO particles, nanospheres had the strongest effect on apoptosis, followed by ZnO nanorods, but significantly lower compared with nanospheres. A zinc acetate solution showed no significant enhancement of the apoptosis rate within the tested concentration range, neither after 2 h (data not shown) nor after 16 h exposure. Thus, the observed induction of apoptosis occurred within a limited concentration range around 20 μg mL^−1^. Lower concentrations of ZnO particles were obviously not sufficient to induce apoptosis in NR8383 cells. An exposure to higher concentrations accompanied by an increase in intracellular Zn^2+^ obviously disrupted apoptosis-related mechanisms. The direct exposure of NR8383 with Zn^2+^ ions supported this assumption.

**Fig. 10:**
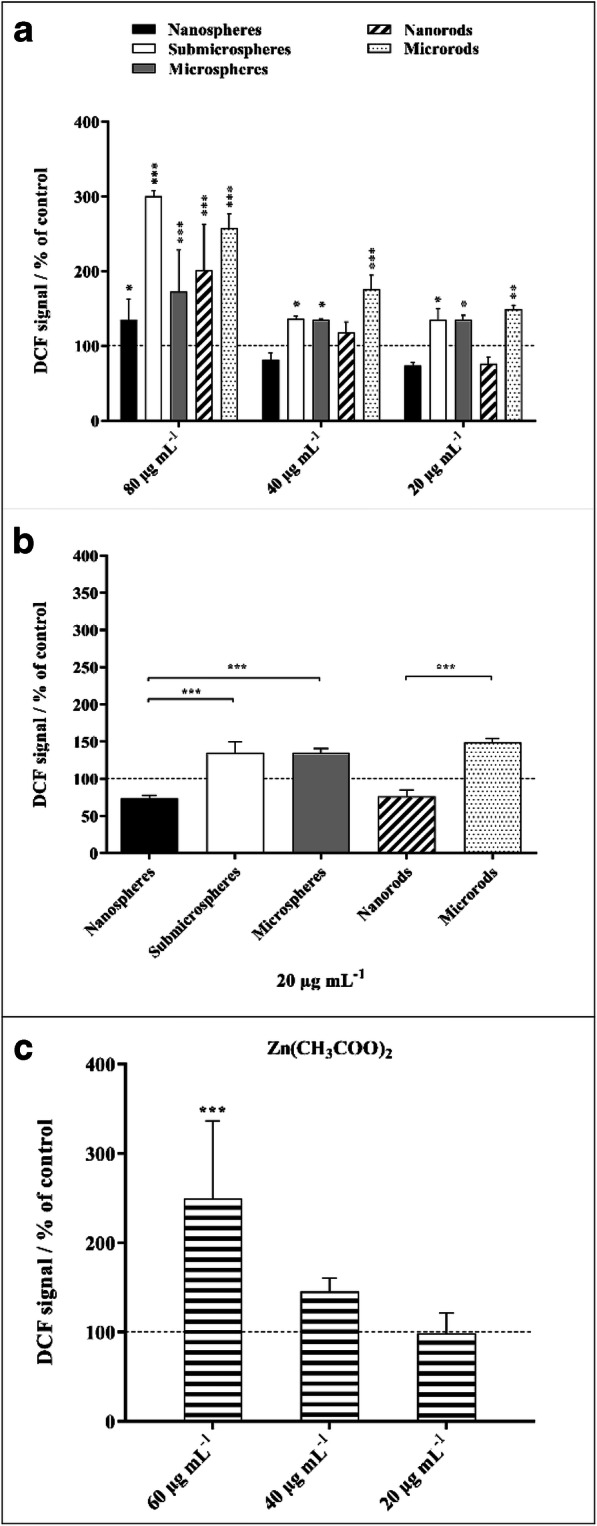
Generation of ROS in NR8383 alveolar macrophages after 2 h of exposure to different ZnO particles (**a**, **b**) and zinc acetate solution as control (**c**). ROS generation was assessed by the mean fluorescence intensity of dichlorofluorescein (DCF) using flow cytometry **(a**). Comparison of the effects of different particle concentrations and morphologies. Comparison of the effects of different particle morphologies at a particle concentration of 20 μg mL^−1^ (**b**). All concentrations refer to solid ZnO, except for zinc acetate where the concentration of Zn^2+^ is given (**c**). Data are expressed as mean ± SD (*N* = 3), given as the percentage of the control (100%, untreated cells). Asterisks (*) indicate significant differences in comparison to the control (*p ≤ 0.05, **p ≤ 0.01, ***p ≤ 0.001)

The generation of ROS by NR8383 macrophages was analyzed after 2 h of particle exposure using the DCFDA cellular ROS Assay Kit and flow cytometry (Fig. 10). Significantly enhanced ROS levels in comparison to untreated cells were observed for all examined ZnO particles after 2 h of incubation at a particle concentration of 80 μg mL^−1^ (zinc acetate 60 μg mL^−1^). Below this concentration, submicrospheres, microspheres, and microrods induced partly elevated ROS levels. Here, microrods provoked the highest effects, which may explain the higher cytotoxicity observed in comparison to microspheres. However, compared with the submicro- and microparticles, nanoparticles exhibited the lowest effects on ROS generation. Comparable with zinc acetate, which led to enhanced ROS formation only at a relatively high concentration of 60 μg mL^−1^.

**Fig. 11:**
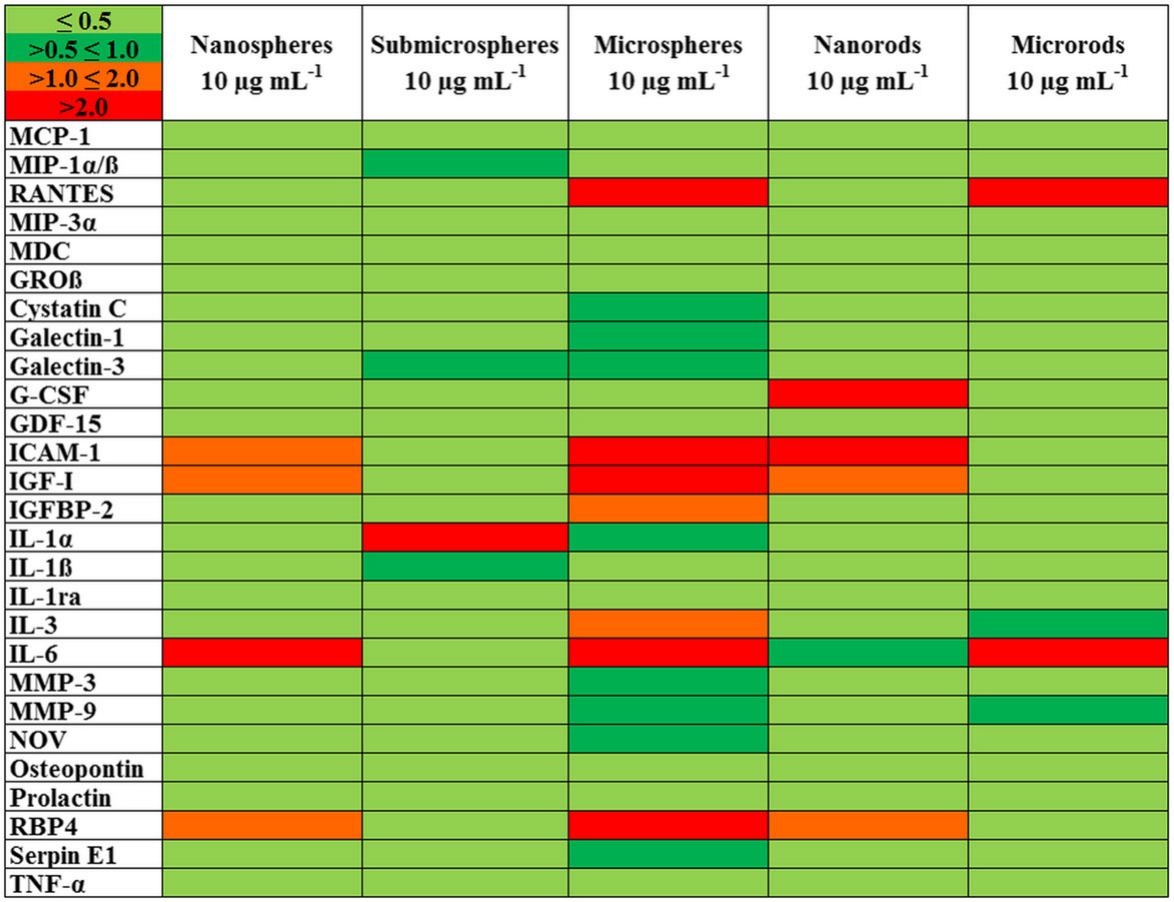
Heat map of 27 bioactive factors of the proteomic repertoire of NR8383 alveolar macrophages after 16 h of exposure to 10 μg mL^−1^ of different ZnO particles obtained by protein microarrays. Each row represents the expression of one factor relative to the minimum and maximum of all values as encoded by the color scale shown in the upper left corner. The intensity scale of the standardized expression values ranges from ≤ 0.5 (green: low expression) to > 2 (red: high expression). All concentrations refer to solid ZnO

As several reports showed an inflammatory activity of ZnO particles in vitro and in vivo (Hsiao and Huang 2011; Monsé et al. 2019; Sahu et al. 2014; Palomäki et al. 2010; Landsiedel et al. 2014a), we analyzed the expression of different bioactive factors by NR8383 macrophages in the presence of ZnO particles. For a qualitative analysis, a chemiluminescent protein microarray was used. Cell culture supernatants were collected after 16 h of particle exposure (10 μg mL^−1^), and 79 signaling molecules, including different cytokines, chemokines, and growth factors, were detected simultaneously. The qualitative heatmap shows the relative expression of 27 of the 79 detected factors selected for a detailed analysis based on the proteomic repertoire of NR8383 cells as reported by Duhamel et al. (Fig. 11) (Duhamel et al. 2015). For all examined ZnO particle types, mainly suppressive effects on the expression of the different factors were observed, and no specific size- or morphology-dependent differences were found. Similar results were obtained for 5 μg mL^−1^ zinc acetate solution (data not shown), indicating a possible Zn^2+^ involvement.

For the subsequent quantitative Sandwich-ELISA analyses, four commonly examined bioactive factors were chosen, namely the pro-inflammatory cytokine interleukin-1β (IL-1β), the growth differentiation factor-15 (GDF-15), the tumor necrosis factor-α (TNF-α), and the chemokine CXCL1 (rat functional homolog of human IL-8). Sandwich-ELISA tests were performed with particle-exposed cell supernatants (5 to 10 μg mL^−1^, 16 h), but no statistically significant effects on the release of these factors were observed (data not shown).

**Fig. 12:**
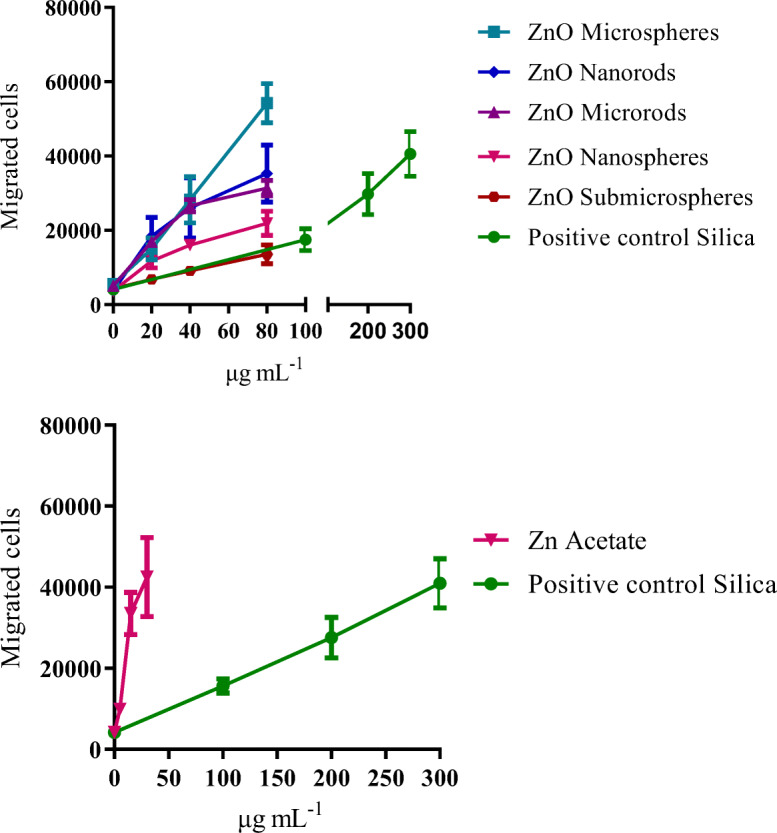
Chemotaxis (migrated cells) of unexposed dHL-60 cells in response to NR8383 cell supernatants that were obtained by incubation with increasing concentrations of ZnO particles (top) and zinc acetate (bottom). Data are expressed as mean ± SD (*N* = 3). Commercially available silica nanoparticles served as positive control. All concentrations refer to solid ZnO or silica material, except for zinc acetate where the concentration of Zn^2+^ is given

The PICMA showed different effects of the various ZnO particles. The strongest chemotaxis was induced by supernatants that were obtained after an incubation with ZnO microspheres, followed by ZnO-nanorods, microrods, nanospheres, and submicrospheres. All investigated ZnO particles acted more strongly in the PICMA compared with the silica-positive control, with the exception of the ZnO submicrospheres. Zinc acetate induced the strongest effects of all investigated compounds, underscoring the effect of dissolved zinc ions. In order to calculate a continuous course of the various ZnO particles, four-parameter log-logistic models were fitted for each type, i.e., ZnO nanorods and microrods, as well as ZnO nanospheres, microspheres, and submicrospheres. The small number of data points at each dose led to a high variation. Nevertheless, the model courses and especially the EC_50_ values indicated a tendency for the real courses. The following 50% effective concentrations (EC_50_) were calculated for the different ZnO particles based on separately adjusted four-parameter log-logistic models: MR 23 ± 3.8 < NR 73 ± 668 < NS 145 ± 1013 ≈ MS 153 ± 865 < SMS 369 ± 1475 μg mL^−1^, respectively (Fig. 12).

## Discussion

As demonstrated above, ZnO particles release Zn^2+^ into their environment, especially under acidic conditions. It is assumed that similarly to silver and copper ions (Loza et al. 2014; Kent and Vikesland 2016), zinc ions interact with different cell components (enzymes, lipids, DNA), disrupting the homeostasis or generating oxidative stress, and causing cellular damage (Sirelkhatim et al. 2015; Liu et al. 2016). In principle, the enhanced toxicity of ZnO nanoparticles compared with submicro- and microparticles can be explained by an enhanced Zn^2+^ release due to the increased surface area and the resulting higher reactivity (Jeevanandam et al. 2018). Although our in vitro dissolution experiments did not show significant differences between the five particle types (probably due to a limited sensitivity of the assay), the cytotoxic effects of ZnO nanoparticles were observed already at particle concentrations of 5 and 10 μg mL^−1^, comparable with an ionic zinc acetate solution. In general, the dissolution behavior of soluble particles in cells as well as the efficiency of cellular particle uptake depends on many intrinsic and extrinsic factors, such as the physicochemical particle properties (e.g., size, shape, surface area, functionalization) or the nature of the environment (e.g., cell type, pH, temperature) (Yu et al. 2011; Patel et al. 2019; Feliu et al. 2017; Behzadi et al. 2017; Messerschmidt et al. 2016).

Smaller ZnO particles were reported to be taken up faster by cells than bigger ones (Verma et al. 2017). A higher toxicity of smaller ZnO particles compared with larger ones was reported (Yu et al. 2011; Hsiao and Huang 2011; Sahu et al. 2014). The fact that rod-shaped ZnO particles had a higher toxicity than spherical ones suggests that both particle size and shape contribute to the toxicity. It should also be noted that for zinc ions, transporter proteins regulate the influx and efflux of zinc ions and that the intracellular zinc concentration is tightly controlled, typically by binding of zinc to proteins (Colvin et al. 2010).

In general, apoptosis can be induced by a variety of stimuli via the extrinsic pathway including death receptor activation and via the intrinsic pathway by activation of caspase cascades upon damage or dysfunction of different cellular components (Mohammadinejad et al. 2019). Particle-induced apoptosis is often associated with generation of ROS resulting in oxidative stress, followed by cellular damage and activation of the intrinsic apoptosis pathway (Mohammadinejad et al. 2019; Kim and Ryu 2013). The induction of apoptosis by ZnO particles was reported earlier (Liu et al. 2016; Mohammadinejad et al. 2019), but the influence of the physicochemical particle properties is not yet understood. For partially soluble particles, both dissolved and particulate fractions may be involved in the biological responses (Ziglari et al. 2020). However, our results demonstrated that dissolved zinc ions did not induce apoptosis, indicating a particle-dependent mechanism. Similarly, Ahamed et al. reported higher apoptotic activity of ZnO nanoparticles on human alveolar carcinoma cells compared with dissolved zinc ions (Ahamed et al. 2011).

Remarkably, ZnO nanospheres did not induce increased ROS levels within the examined concentration range, although they exhibited the highest apoptotic activity among the tested particles. It is known that a particle-related apoptosis is often associated with the generation of ROS and the resulting oxidative damage of cell components, especially in the case of nanoparticles (Ahamed et al. 2011; Shrivastava et al. 2014; Liu et al. 2016). Our results indicate that the examined ZnO nanoparticles induced other pathways of apoptosis, e.g., by activation of the death receptor, by ER stress, and/or by mitochondrial damage (Mohammadinejad et al. 2019). Actually, some studies reported an ROS-independent apoptosis induction by ZnO particles (80 to 250 nm) by causing mitochondrial dysfunction in response to zinc stress as well as a general metabolic perturbation, leading to DNA damage even at absent or low oxidative stress responses (Chevallet et al. 2016; Triboulet et al. 2014). Remarkably, Verma et al. reported ROS quenching for ZnO nanoparticles (50 to 60 nm), together with a strong apoptotic activity (Verma et al. 2017).

The reported correlation between ROS and apoptosis is generally attributed to the soluble Zn^2+^ fraction. However, our results demonstrate that zinc acetate led to increased ROS formation only at high concentrations and did not induce apoptosis at all. Thus, the observed apoptotic activity of ZnO particles is mainly related to the particulate fraction. Similar particle-induced effects have been observed by Seong and Lee where enhanced DNA fragmentation and mitochondrial dysfunction were induced by non-soluble gold nanoparticles (30 nm) in the absence of an oxidative stress response (Seong and Lee 2018).

In general, toxic ions released by soluble particles in the acidic environment of lysosomes can accumulate in these organelles and may cause lysosome destabilization and inflammation (Donaldson et al. 2013). In contrast, our results suggest that the examined ZnO particles have immunosuppressive properties. Similar to our findings, Kim et al. reported immunosuppressive effects in vitro (RAW264.7 cells) and in vivo (C57BL/6 mice) for spherical ZnO nanoparticles (30–90 nm) (Kim et al. 2014). In an inhalation study of Adamcakova-Dodd et al. in C57Bl/6 mice, ZnO nanoparticles (10–30 nm) induced only minimal pulmonary inflammation or lung histopathological changes (Adamcakova-Dodd et al. 2014).

Table 4 summarizes all cell-biological data described above. It is clear that there is no easy way to bring the effects into a consistent order, indicating a multifactorial network of mechanisms which regulates the biological response to zinc oxide nanoparticles. As the cellular actions of zinc ions are manifold due to the multitude of zin ion-binding proteins (Colvin et al. 2010), it is no surprise that the effects of zinc ions after particle uptake and dissolution are complex.

**Table 4:** Summary of the cell-biological effects of all ZnO particles and zinc ions as control. *NS* nanospheres, *SMS* submicrospheres, *MS* microspheres, *NR* nanorods, *MR* microrods, *Zn*^*2+*^ zinc ions (zinc acetate)

Particle-cell contact	MR ≈ MS > SMS > NR ≈ NS
Intracellular zinc ion release	NS ≈ NR ≈ MS > SMS ≈ MR
cytotoxicity	Zn^2+^ ≈ NS ≈ NR > MR > SMS ≈ MS
Apoptosis induction	NS > MS ≈ NR ≈ MR > SMS ≈ Zn^2+^
Cell activation (ROS)	MR > SMS ≈ MS > NS ≈ NR ≈ Zn^2+^
Cytokine release	Zn^2+^ ≈ MR ≈ SMS ≈ MS ≈ NS ≈ NR
PICMA	Zn^2+^ > MR > NR > NS ≈ MS > SMS

## Conclusions

In summary, the targeted synthesis of zinc oxide particles relevant to occupational medicine made it possible for the first time to assess the biological effects of well-dispersed chemically identical particles which differ only in size and shape. The five types of zinc oxide nanoparticles induce different effects on NR8383 macrophages, but not all results are coherent. Despite the fact that the dissolution rate of the particles as measured in vitro is almost identical, their cell-biological effects are different. We assume that this is due to different mechanisms which are triggered by the uptake kinetics into cells and the kinetics of the intracellular dissolution and the subsequent release of zinc ions. This is supported by the observation that dissolved zinc ions sometimes have a strong effect (cytotoxicity, PICMA) and sometimes a weak effect (apoptosis induction, ROS generation). We conclude that there are different mechanisms for cell-biological effects that depend on the particle characteristics, of which size and shape are only two.

## Methods

### Chemicals

We have used zinc nitrate hexahydrate (Zn(NO_3_)_2_·6 H_2_O, Alfa Aesar, 98%), zinc acetate dihydrate (Zn(CH_3_COO)_2_·2 H_2_O, Alfa Aesar, > 98%), poly(N-vinylpyrrolidone) (PVP K 55; Sigma-Aldrich, p.a., *M* = 55,000 g mol^−1^), diethylene glycol (DEG, Sigma-Aldrich, 99%), ethylene glycol (EG; Sigma-Aldrich, 99.8%), dimethylformamide (DMF; Fischer Chemicals, > 99.5%), and ultrapure water (Purelab ultra instrument from ELGA). All chemicals were used as obtained without further purification. Before the experiments, all glassware was cleaned with boiling aqua regia and twice with boiling ultrapure water. Finally, all glassware was sterilized at 200 °C for 3 h. All synthesized particles were purified by centrifugation with a Heraeus Fresco 21 centrifuge (Thermo Scientific).

### Synthesis of ZnO nanoparticles

PVP-coated ZnO nanorods were prepared by a polyol method according to Lee et al. (2008) with slight modifications. Zinc acetate dihydrate (8.78 g), PVP (0.233 g), and 4.32 mL water were added to 96 mL of diethylene glycol (DEG) and stirred for 10 min at room temperature. Then, the reaction mixture was heated under vigorous stirring to 180 °C. The solid zinc acetate had completely dissolved at 120 °C. The reaction mixture was stirred for 30 min at 180 °C and then quenched to room temperature in an ice bath. The nanoparticles were purified by triple centrifugation (3500 rpm, 60 min) and redispersion in ethanol and then dried at 80 °C for 4 h. For the synthesis of PVP-coated ZnO nanospheres, the solvent was changed to ethylene glycol (EG). All other parameters were the same as with the nanorods.

### Synthesis of ZnO microparticles

A one-pot synthesis of PVP-coated ZnO microspheres in DMF was developed based on the method reported by Yao et al. (Yao and Zeng 2007). 1.485 g of zinc nitrate hexahydrate and 3 g of PVP were completely dissolved in 200 mL of DMF under vigorous stirring at room temperature. After stirring for 10 min at room temperature, the solution was rapidly heated to 100 °C. After 20 min, the reaction mixture had assumed a turbid color, indicating a nucleation of ZnO particles. After another 20 min, the reaction mixture was heated to 120 °C and stirred for 2 h. Finally, the mixture was quenched to room temperature with an ice bath. The particles were collected by centrifugation (3500 rpm, 30 min), washed with ethanol several times, and dried at 80 °C for 4 h. A one-pot synthesis of PVP-coated ZnO microrods was performed by adding water to DMF in a volume ratio of 5 mL:95 mL. All other parameters remained the same.

### Synthesis of ZnO submicroparticles

The synthesis of PVP-coated ZnO submicroparticles was performed in the same way as the synthesis of PVP-coated ZnO microspheres, but in this case, the reaction time was shortened. Without changing the concentration of reactants, the reaction mixture was heated to 120 °C and the reaction time was reduced from 120 to 20 min. After synthesis, the oil bath was removed and quickly replaced by an ice bath. Purification of the particles was performed by triple centrifugation (3500 rpm, 30 min) and redispersion in ethanol, followed by drying of the particles at 80 °C for 4 h.

### In vitro dissolution tests

Ten milligrams of PVP-coated ZnO particles were redispersed in 50 mL of four different media: ultrapure water, RPMI medium (Gibco, supplemented with 10% fetal bovine serum, FBS), simulated lysosomal medium, and citrate-free acetate buffer.

The simulated lysosomal medium (pH = 4.5) was prepared according to (Henderson et al. 2014). We used sodium chloride (NaCl, 3.21 g L^−1^, Bernd Kraft, > 99.5%) sodium hydroxide (NaOH, 6.0 g L^−1^, Baker, 99%), citric acid (20.08 g L^−1^, Fluka, > 99.5%), calcium chloride dihydrate (0.097 g L^−1^, GrisChem, > 99%), sodium phosphate dibasic heptahydrate (Na_2_HPO_4_·7 H_2_O, 0.179 g L^−1^, Riedel-de Haën, 99%), sodium sulfate heptahydrate (Na_2_SO_4_·10 H_2_O, 0.039 g L^−1^, Fluka, 99%), magnesium chloride hexahydrate (MgCl_2_·6 H_2_O, 0.106 g L^−1^, GrisChem, 99%), glycine (0.059 g L^−1^, Biomol, > 99%), sodium citrate dihydrate (0.077 g L^−1^, Sigma-Aldrich, 99%), sodium hydrogen *L*-tartrate (0.090 g L^−1^, Alfa Aesar, 98%), sodium *L*-lactate (0.085 g L^−1^, Sigma-Aldrich, >99%), sodium pyruvate (0.85 g L^−1^, Sigma-Aldrich, >99%), and formaldehyde (0.3 mL L^−1^, Fluka, p.a.). The solution was filled with water to 300 mL. The citrate-free acetate buffer (pH = 4.8) was prepared with an aqueous solution of acetic acid (300 mL, 1 mol L^−1^, Carl Roth) and sodium acetate trihydrate (1.22 mol L ^−1^, Sigma-Aldrich) instead of citric acid/sodium citrate. All other compounds were the same as above.

The particle dispersion (10 mg in 50 mL medium) was placed into a closed round bottom flask (200 mL) and stirred at 25 °C (water) or at 37 °C (RPMI/FCS) for 5 days under sterile conditions. After 30 min and then after each day, 1 mL of the particle dispersion was taken and filtered (nanoparticles: inorganic membrane filter, Whatman Anotrop 10 Plus; 0.02 μm; submicro- and microparticles: inorganic membrane filter, Whatman Anotrop 25; 0.2 μm) to separate zinc ions from ZnO particles. When the particle dispersion was taken, its pH was measured (pH meter HANNA HI 991001). The pH increased slightly with time due to the dissolution of zinc oxide (in water: 6.9 to 7.9, RPMI/FCS: 6.9 to 7.6). For the dissolution tests of ZnO particles in simulated lysomal media, the particle solution was stirred only for 1 h due to the rapid dissolution at pH = 4.5 (citrate-buffered) and pH = 4.8 (acetate-buffered). Finally, the zinc content in the isolated particles and the zinc ion concentration in the filtrates were determined by AAS.

### Cell culture

The biological effect of the particles was studied with the cell line NR8383 (rat alveolar macrophages, LGC Standards GmbH, Wesel, Germany). The cells were cultivated in Ham’s F12 medium containing 15% fetal calf serum (FCS, GIBCO, Invitrogen, Karlsruhe, Germany) in 175 cm^2^ cell culture flasks (BD Falcon, Becton Dickinson GmbH, Heidelberg, Germany) at standard cell culture conditions (humidified atmosphere, 37 °C, 5% CO_2_). The NR8383 cells were only partly adherent. The ratio between adherent and non-adherent cells was about 1:1. For cell experiments, adherent cells were detached from the cell culture flasks with a TPP cell scraper (TPP Techno Plastic Products AG, Trasadingen, Switzerland), subsequently combined with non-adherent cells, and seeded into 24-well cell culture plates (BD Falcon) at a concentration of 2.4 × 10^5^ cells cm^−2^.

### Intracellular zinc ion concentration

The intracellular concentration of zinc ions after 2 h of exposure of NR8383 cells to different ZnO particles at various concentrations (80, 40, 20 μg ZnO mL^−1^) was measured with the Zn^2+^-selective indicator FluoZin-3 (Invitrogen) and flow cytometry. After incubation with the ZnO particles, the cells were collected in 5-mL tubes (BD Biosciences) as described above and stained with 100 μM of FluoZin-3 for 30 min at room temperature. For the discrimination of non-viable cells, staining with 50 μg mL^−1^ propidium iodide (PI, Sigma-Aldrich, Taufkirchen, Germany) was also performed (10 min, room temperature).

### Cytotoxicity assay

The cytotoxicity of different ZnO particles at various concentrations (80, 40, 20, 10, 5 μg ZnO mL^−1^) for NR8383 cells was analyzed by flow cytometry. A solution of zinc acetate (Alfa Aesar, Karlsruhe, Germany, 98%) was used as control (40, 20, 15, 5 μg Zn^2+^ mL^−1^). After 16 h of particle or zinc acetate exposure, adherent and non-adherent cells were combined in 5 mL as described above. Non-viable cells were labeled with 50 μg mL^−1^ PI for 10 min at room temperature. The number of non-viable cells (PI positive) was determined by flow cytometry.

### Apoptosis assay

The induction of apoptosis in NR8383 cells by ZnO particles was investigated with the Annexin V apoptosis assay and flow cytometry. The cells were incubated for 16 h with ZnO particles (80, 40, 20, 10, 5 μg ZnO mL^−1^) as well as with zinc acetate solution (40, 20, 10, 5 μg Zn^2+^ mL^−1^). After incubation, adherent and non-adherent cells were combined in 5 mL tubes as described above. Staining of early apoptotic cells was performed with FITC-conjugated Annexin V (BioLegend GmbH, Koblenz, Germany) according to the manufacturer’s protocol in Annexin V Binding Buffer containing CaCl_2_ and MgCl_2_ (BioLegend GmbH), while necrotic and late apoptotic cells with damaged membranes were excluded by counterstaining with 50 μg mL^−1^ of PI (15 min, room temperature).

### Generation of reactive oxygen species

The formation of ROS in NR8383 cells after 2 h of incubation with 80 μg ZnO mL^−1^ of different ZnO particles was investigated qualitatively with the cell-permeant ROS indicator CellROX Green (Thermo Fisher Scientific, Waltham, USA) which gives a strong fluorescence after oxidation. After particle exposure, the cells were stained with 5 μM CellROX Green for 30 min under cell culture conditions and analyzed by confocal laser scanning microscopy. Cells exposed to 100 μM H_2_O_2_ (Sigma-Aldrich) for 30 min under cell culture conditions served as a positive control for elevated ROS levels.

The quantitative analysis of ROS formation was performed by the DCFDA assay with flow cytometry. Cells were incubated with different concentrations of ZnO particles (80, 40, 20 μg ZnO mL^−1^) as well as a zinc acetate solution (60, 40, 20 μg Zn^2+^ mL^−1^) for 2 h under cell culture conditions. Next, 20 μM of the cell-permeant ROS indicator 2′,7′-dichlorodihydrofluorescein diacetate (H_2_DCFDA, Thermo Fisher Scientific) was added, and the cells were incubated for 30 min at 37 °C. The non-fluorescent H_2_DCFDA diffuses into cells, where it is deacetylated by intracellular esterases and converted to highly fluorescent 2′,7′-dichlorofluorescein (DCF) by oxidation. For discrimination between viable and non-viable cells, an additional PI staining was performed (50 μg mL^−1^, 10 min, room temperature).

### Protein microarray

After incubation of NR8383 cells with different ZnO particles (10 μg ZnO mL^−1^) for 16 h, the supernatants were collected and centrifuged at 300*g* for 10 min and stored at − 20 °C until microarray analysis (Profiler Array Rat XL Cytokine Array Kit, Bio-Techne GmbH, Wiesbaden-Nordenstadt, Germany). The assay detected 79 different cytokines, growth factors, and other mediators and permitted a semi-quantitative analysis. The membrane-based sandwich immunoarray consisted of a nitrocellulose membrane on which the capture antibodies were spotted as duplicated dots. The target proteins in the sample were bound to the capture antibodies and detected with biotinylated detection antibodies, followed by visualization with chemiluminescent detection reagents. For analysis, the manufacturer’s instructions were observed and the chemiluminescence signals were detected and quantified by a microarray imager and the ImageQuantTL software (Amersham Imager 600 RGB, GE Healthcare Bio-Science, Uppsala, Schweden). For the subsequent detailed analysis, 27 factors were selected based on the proteomic repertoire of NR8383 cells (Duhamel et al. 2015).

Quantitative analyses were performed with cell culture supernatants after 16 h of exposure to ZnO particles (5 to 10 μg ZnO mL^−1^) with Sandwich-ELISA Kits (R&D Systems Quantikine, Bio-Techne GmbH, Wiesbaden, Germany).

### PICMA

NR8383 cells were cultivated at 37 °C, 100% humidity, and 5% CO_2_ in Ham’s F-12 + 15% FCS medium (Biochrom KG, Berlin, Germany), 2 mM l-glutamine, 100 g L^−1^ penicillin, and 100 U mL^−1^ streptomycin. In 25 mL (175 cm^2^) medium, approximately 3 × 10^6^ cells were seeded.

HL-60 cells were obtained from DSMZ (Braunschweig, Germany). *Trans*-retinal differentiated HL-60 cells (dHL-60) were used to induce the chemotaxis. For this, the HL-60 cells were cultivated for 3 days in RPMI 1640 medium (Biochrom), 10% FSC, 2 mM l-glutamine, 100 g L^−1^ penicillin, 100 U mL^−1^ streptomycin, and 1 μM *trans*-retinoic acid at 37 °C, 100% humidity and 5% CO_2_ (Breitman et al. 1980). In conventional culture dishes, the dHL-60 cells grow adherent.

For the particle-induced cell migration assay, we suspended NR8383 rat macrophages (3∙10^6^ cells mL^−1^) with a vortex in 1 mL Ham’s F-12 medium containing 15% FCS, 2 mM l-glutamine, 100 mg L^−1^ penicillin, and 100 U mL^−1^ streptomycin. Then, the cells were seeded in 12.5-cm^2^ cell culture flasks to a final volume of 3 mL (2.4 × 10^5^ cells cm^−2^). Note that it is also possible to perform the assay in a smaller volume at constant surface to volume ratio.

As negative control, we used a sample of cells without particles. We repeated the subsequent experiments up to the concentrations which gave the maximum induction of chemotaxis. The cells were incubated with the particles for 16 h at 37 °C, 100% humidity, and 5% CO_2_. Afterwards, we removed the cells by centrifugation at 300*g* for 5 min. The particles were removed by centrifugation at 15,000*g* for 10 min at room temperature. We used the supernatants immediately thereafter for the cell migration tests.

We investigated the cell migration according to Boyden (1962), but with the modifications described earlier by Westphal et al. (2015) and Schremmer et al. (2016). For this, we exclusively applied permanent cell lines in the following way. We added 2 × 10^5^ unchallenged dHL-60 cells to 200 μL RPMI 1640 medium without FCS and seeded the cells in each plate well insert (THINCERT, 3-μm pore size, Greiner bio-one, Frickenhausen, Germany) and placed the insert into the cavities of 24 black well plates (Krystal, Dunn Labortechnik, Asbach, Germany). A total of 500 μL of the supernatants of the particle-incubated NR8383 cells was added to the lower chamber. The migration of dHL-60 cells across the membrane was observed for 24 h at 37 °C, 100% humidity, and 5% CO_2_. 10^5^ HL-60 cells were seeded directly into four-plate wells that were left without inserts for calibration.

Calcein-AM was used to stain migrated cells. Cell calibration was performed for 60 min at 37 °C, 5% CO_2_ and 100% humidity by adding 500 μL calcein-AM to the plate wells (> 90% HPLC, Sigma-Aldrich, Steinheim, Germany). Calcein-AM was used as 4 mM solution in DMSO, stored in aliquots at − 18 °C, and diluted to the final concentration of 4 μM in PBS.

After that, the cell suspensions were removed from the plate wells and collected by centrifugation at 300*g* for 5 min at room temperature. The cells were re-suspended in 150 μL while 850 μL of the supernatant was discarded. Furthermore, the adherent cells at the outside of the inserts were detached with 500 μL trypsin/EDTA (0.05%/0.02%, Biochrom) for 10 min at 37 °C, 5% CO_2_, and 100% humidity. Then, the inserts were removed from the plate wells. The 150 μL containing the collected cells were added to the plate wells that contained 500 μL of trypsin/EDTA-detached cells. Cell counting was done by fluorescence spectroscopy at 490/520 nm and related to the cell calibration (SpectraMax M3, Molecular Devices, Sunnyvale, USA).

In terms of statistical significance, acceptance criteria for a valid test were positive control (nanosized silica) and negative control within the range of the established controls as established in our laboratory. In this context, we defined a positive response as a dose-dependent increase of cell migration across at least two consecutive concentrations, with a maximum that exceeded the base rate by at least twice the highest concentration (Westphal et al. 2015).

As reference compound, we used a silica reference sample (CAS No. 7631-86-9, Lot MKBF2889V, 99.5%, 10–20 nm; Sigma-Aldrich, Steinheim, Germany). These particles were previously characterized in detail and consisted of agglomerated X-ray amorphous silica particles with a primary particle size of about 50 nm and an agglomerate size of about 2 μm (Westphal et al. 2015).

### Instruments

Dynamic light scattering for particle size analysis and zeta-potential determination were carried out with a Malvern Zetasizer Nano ZS ZEN 3600 instrument (Malvern Panalytical Ltd.; 25 °C, laser wavelength 633 nm). The light scattering was monitored at a fixed angle of 173° in backward scattering mode. The peak profile of the size distribution was analyzed by a log-normal distribution fit. The average diameter was taken as the mean value of the maximum of the size distribution *x*_*c*_ from the log-normal distribution fit analysis and the empirical standard deviation.

Ultraviolet-visible spectroscopy (UV/vis) was performed with a Varian Cary 300 instrument (Agilent Technologies, Inc.). Suprasil® quartz cuvettes with a sample volume of 3 mL were used after dilution and background correction.

The ZnO particles were dissolved in concentrated nitric acid before the atomic absorption spectroscopy (AAS). AAS was carried out with a Thermo Electron M-Series instrument (Thermo Fisher Scientific) according to DIN EN lSO/lEC 17025:2005.

Thermogravimetric analysis (TGA) was performed with a Netzsch STA 449 F3 Jupiter instrument to determine the content of coating polymer in the samples. The purified and dried particles were heated in an open alumina crucible with 5 K min^−1^ from 20 to 1000 °C under dynamic oxygen atmosphere.

Scanning electron microscopy of the particles was performed with a FEI ESEM Quanta 400 FEG microscope. Prior to the SEM investigation, the samples were sputter-coated with a thin conductive AuPd 80:20 layer.

For X-ray powder diffraction (XRD), the particle dispersion was shock-frozen with liquid nitrogen and lyophilized at 0.31 mbar and − 10 °C in a Christ Alpha 2-4 LSC instrument. XRD measurements were performed with a Bruker D8 Advance instrument in Bragg-Brentano geometry with Cu Kα radiation (*λ* = 1.54 Å, 40 kV and 40 mA) with a single-crystalline silicon sample holder in the crystallographic (911) plane to minimize scattering. The powder samples were investigated from 5 to 90° 2Θ with a step size of 0.01° and a counting time of 0.6 s at each step. The instrumental peak broadening was determined with lanthanum hexaboride (LaB_6_) from NIST (National Institute of Standards and Technology; reference compound) as internal standard. Rietveld refinement was performed with the program package TOPAS 5.0 from Bruker to determine the lattice parameters (*a* and *c*), the isotropic and anisotropic crystallite size (*D* and *D*_*A*_), and the microstrain (*ε*). For the calculation of *D*, the Scherrer and Stokes-Wilson equations were used (Klug and Alexander 1974). The diffraction pattern of hexagonal zinc oxide was taken from the ICDD database (International Centre of Diffraction Data) as reference (#36-1451) and used for the qualitative phase analysis with Diffrac.Suite EVA V1.2 (Bruker).

Flow cytometric analyses were carried out with an FACSCalibur flow cytometer (BD Bioscience, Heidelberg, Germany). For each measurement, 10,000 cells were analyzed, and the data were quantified with the CELLQuest 1.2.2 software (BD Biosciences).

Confocal laser scanning microscopy was performed with a Zeiss LSM 700 instrument (Carl Zeiss Microscopy GmbH, Jena, Germany). Fluorescence images were taken (Zeiss LSM 700 microscope and Zen 2010 software) and digitally processed using Adobe Photoshop 7 (Adobe Systems GmbH, CA, USA).

The number of particles in 1 g of solid material was computed from the average particle mass of one sphere and one rod, respectively:

$$ {\displaystyle \begin{array}{c}m\mathrm{spheres}=\frac{4}{3}\ \uppi\ r3\ \uprho \\ {}m\mathrm{rods}=\pi\ {r}^2L\ \rho \end{array}} $$

with *r* the particle radius and *L* the particle length, both obtained from SEM (Table 1), and *ρ* the density of ZnO (4030 kg m^3^). The specific surface areas of the particles (m^2^ g^−1^) were computed as follows:

$$ {\displaystyle \begin{array}{c}S\mathrm{spheres}=4\uppi\ {r}^2 Nparticles\  per\ 1g\\ {} Srods=\left(2\pi\ {r}^2+2\pi rL\right)\  Nparticles\  per\ 1g\end{array}} $$

### Statistical analysis

Data are expressed as the mean ± SD (*n* = 3) and given as the percentage of the control (cells not exposed to particles). For statistical evaluation, one-way analysis of variance (ANOVA) with Bonferroni’s multiple comparison test was applied using the GraphPad Prism software (GraphPad Software, Inc., CA, USA), while *p* values ≤ 0.05 were considered statistically significant.

EC_50_ values (PICMA): To illustrate the dose-response relation more precisely, four-parameter log-logistic models were used. According to Van der Vliet and Ritz (Van der Vliet and Ritz 2013), the four-parameter log-logistic model is defined as:

$$ f\left(x,\left(b,c,d,e\right)\right)=c+\frac{d-c}{1+\exp \left(b\left(\log (x)-\log (e)\right)\right)} $$

with *b*, *c*, *d*, and *e* used as corresponding parameters. *b* represents the slope of the curve, *c* indents the lower and *d* the upper asymptote, and *e* is the effective concentration EC_50_.

The R-package drc developed by Ritz et al. (2016) provides specialized analyses for such dose-response relations. Especially, the function drm is used for fitting dose-response models.

## Data Availability

The datasets used and/or analyzed during the current study are available from the corresponding authors on reasonable request.
